# Water distribution pipelines inside the monastery of El Escorial during the sixteenth century

**DOI:** 10.1007/s12685-024-00352-7

**Published:** 2024-11-19

**Authors:** Pablo Gumiel-Campos

**Affiliations:** Universidade Nova de Lisboa, Lisbon, Portugal

**Keywords:** El Escorial, Water, Hydraulics, Pipelines, Philip II, Francisco de Montalbán

## Abstract

This article addresses the study of the internal hydraulic infrastructure of the Royal Monastery of San Lorenzo del Escorial. The vast network of pipes that supplied water to the numerous fountains, tanks, and other devices in the building. The methodology was based on two fundamental pillars: an analysis of the historical sources related to water and a cataloguing of the preserved architectonical remains of the infrastructure. A new sketch of the water supply network of the building has been drawn using ArcGIS Pro software. In doing so, this article provides a more accurate and detailed knowledge of the building's plumbing system.

## Historical contextualisation, state-of-the-art, objectives and methodology

In 1558, Philip II of Spain (1556–1598) planned to found a monastery. The dedicatory letter stated that: Firstly, the monastery was to be a place to glorify and render thanks to God. Secondly, it was where the king’s body and those of his ancestors should rest. Thirdly, it was to be dedicated to Saint Lawrence, martyr of Rome, on whose feast day (10 August 1557) Philip II had won the Battle of St. Quentin (Bermejo 1820, p. 11). Fourthly, it was to be handed over to the Order of St. Jerome, so that, finally, a seminary could be established. The monastery was constructed between 1361 and 1584 in the Sierra de Guadarrama (Madrid) near the village of El Escorial. In 1559, the king commissioned the architect Juan Bautista de Toledo, after whose death in 1567, the works were finished by Juan de Herrera.

It is important to understand El Escorial as a monastery with a palatial wing. As Chueca Goitia has been able to show, the attachment of a palace to an existing charterhouse was a deeply rooted tradition in the mediaeval Iberian Peninsula (1981). The choice of the Hieronymite Order had not been by chance, since it had long been connected to the Iberian Crowns. Monasteries such as Sisla, Lupiana, Guadalupe, Guisando, Lisboa, Madrid or Yuste were closely linked to the monarchy. The interrelation between palace and monastery was also reflected in the plan of the building, which was divided into three individual spaces: the convent (*Domus Sacerdotum*), the temple (*Domus Domini*) and the palace (*Domus Regia*) (Chueca Goitia 1981, p. 47).

At first sight, the building appears to be a dry and large granite building, however, it had an immense water supply network that reached almost all the courtyards and chambers of the complex. The study of this water network is complex, and this fact has been reflected in the historiography. In 1820, Fray Damián Bermejo listed a total of 45 interior fountains and 31 exterior ones in his description of the building (Bermejo 1820, p. 339). This data has been repeated throughout nineteenth and twentieth-century historiography without question. Only around the 1980s did research focused on the water system of the monastery begin to appear. Luís De Castro Caturla (1986, p. 118) made the first approach to the hydraulics of the building; Pedro Criado Juárez (2000) performed an archaeological campaign on the cisterns of the courtyard of Hospedería; ten years ago Pilar Chías Navarro (2013) wrote about the water landscape surrounding the monastery and its sewers; finally, Carmen Toribio Marín (2015, 2016) has researched on the water shapes in the gardens of El Escorial. However, these researchers have not directly addressed the study of the monastery's complex network of internal plumbing distribution. This work offers a more granular view than previously possible.

**Fig. 1:**
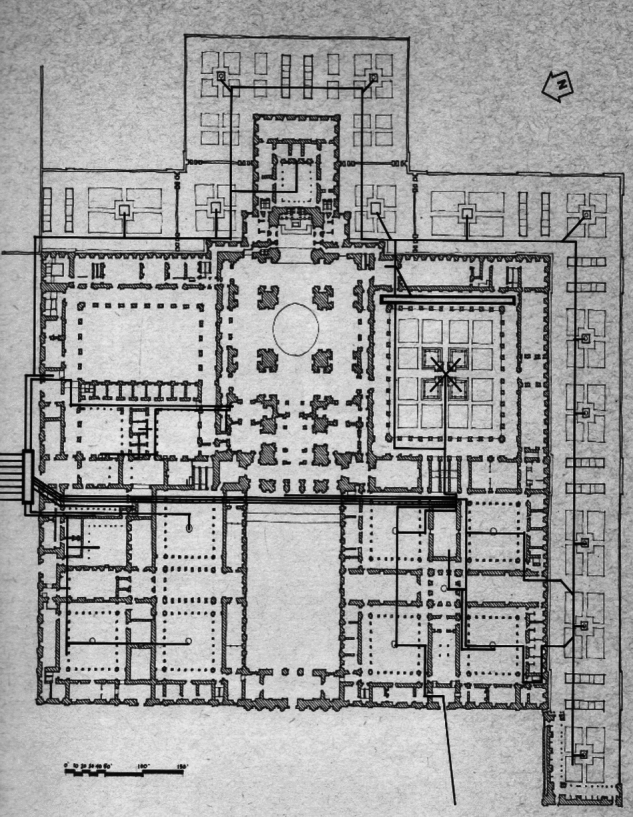
George Kubler (1983). Sketch of the hydraulic network of the monastery of El Escorial

**Fig. 2:**
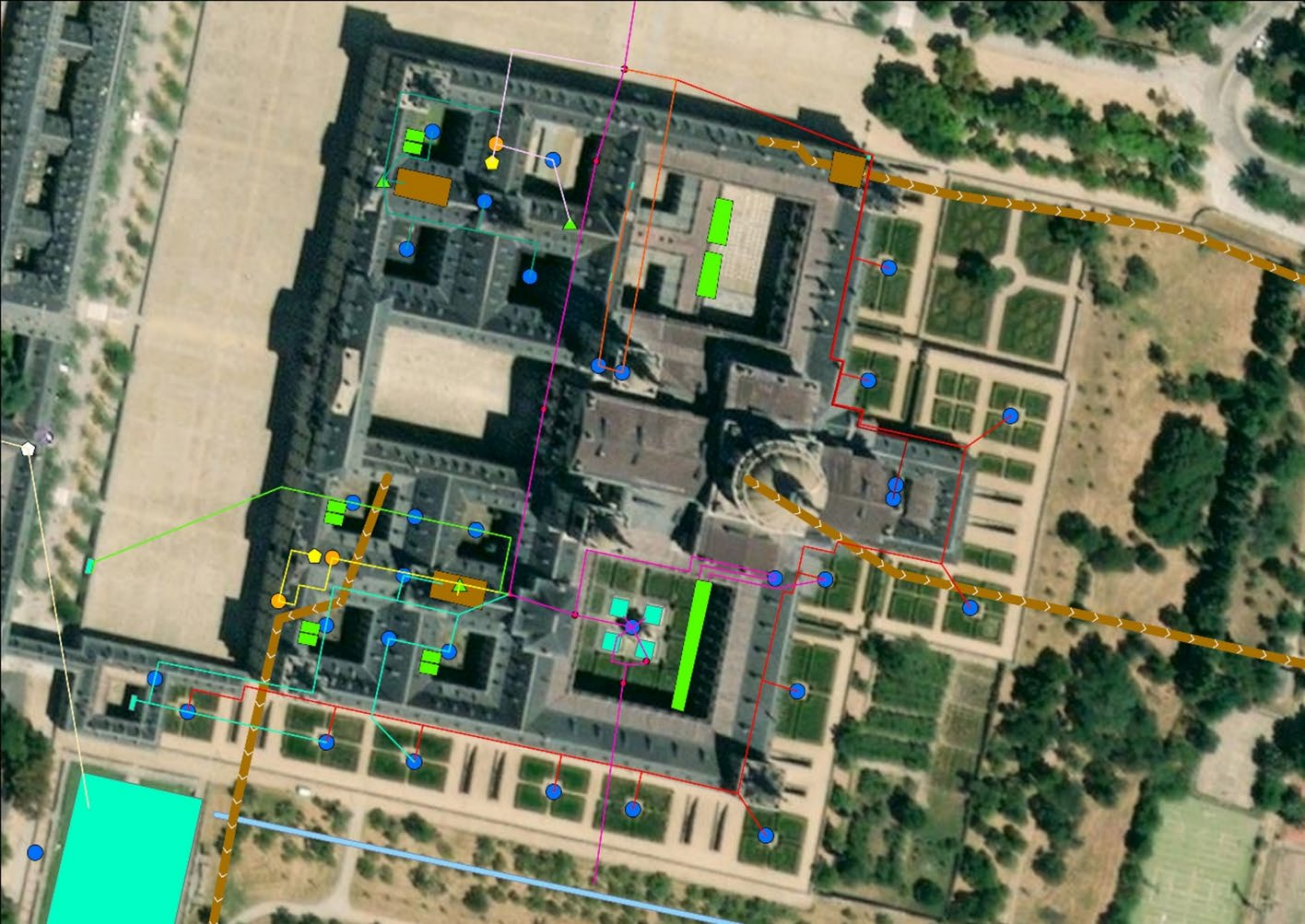
ArcGIS general map of the internal hydraulics of the Escorial Monastery

The situation is even more complicated by the lack of a map of this hydraulic infrastructure. The sketch published by Father Gregorio De Andrés in 1965 (1965) and the copy by George Kubler (1983) must be acknowledged (Fig. 1). However, the accuracy of both drawings is not very comprehensive. Thanks to the actual technology and wanting to exploit the benefits of the digital humanities, a new sketch for the hydraulic distribution network of the building has been drawn using ArcGIS pro software (Fig. 2) (The map can also be consulted and navigated on: https://aquainpalatio.wixsite.com/home/el-escorial [last access: 11.09.2024]). Even so, this map is still not precise enough. The detailed tracing of the building's galleries and canalisations could only be carried out through an archaeological campaign, which in many areas of the monastery is simply impossible to implement.

Therefore, this work has been based on two fundamental pillars: On the one hand, an in-depth analysis of the documentary sources related to the hydraulic system has been carried out. Among all of them, must be highlighted the *Libro de la Fontanería* (De Andrés 1965). This is a report written in 1645, attributable to Fray Nicolas de Madrid, in which a study of the water supply system was made to correct and prevent the flooding of the Monastery´s Pantheon. According to the archivist of the Monastery, the original document of 1645 is lost. It was formerly located in box number 15 under the title *Noticia de todas las fuentes que tiene este Rl. Monasterio de Sn. Lorenzo*. A copy was written in 1792, currently allocated in the archive under the signature Z-IV-16. In 1965, Gregorio de Andrés made a copy of that eighteenth-century copy. I have been able to compare both, and there are no differences. For practical reasons, I will be referring to 1965’s source. Nevertheless, the *Libro de la Fontanería* is not the only source of relevance to this study. The sixteenth and seventeenth-century descriptions by Juan Alonso de Almela (1962), Jean Lhermite (Lhermite 2005), father Sigüenza (2010), and Juan de Herrera (1589), as well as the extensive record of historical sources preserved in the archives, have also been decisive for this study.

On the other hand, this work has been based on the cataloguing and georeferencing of the preserved remains of the hydraulic infrastructure, always within the limitations of the historian. In this respect, I would like to thank Sergio Pascual García and other technicians from Patrimonio Nacional who were kind enough to guide us through the Royal Site.

## The eleven rain cisterns

During the first ten years of the building's life, a very basic hydraulic system of monastic tradition was conceived. It was based on the use of rainwater. For this purpose, eleven large-capacity cisterns were installed to collect the water that ran off the roofs and the courtyards. In Fig. 2, these cisterns are represented as large rectangular areas of light green colour. Their dating is supported by several historical sources. In 1564, Juan Bautista de Toledo wrote a letter certifying the beginning of the works of the cisterns under the minor cloisters (Bustamante García 1994, p. 88). A year later the construction of the main cistern of the Evangelistas cloister began (Bustamante García 1994, p. 110). In another letter dated April 1565, King Philip II sent his opinions on how the cisterns should be laid out^1^ (Portabales Pichel 1945, p. 3). The construction of the cisterns continued until 1569 when a contract was signed to cover the ones of the main courtyard with a stone vault (De Andrés 1972, p. 17).

These eleven cisterns were arranged underground in the vast network of cellars in the monastery. I have not had the opportunity to explore them, but their morphology was studied by Criado Juárez, who was able to analyse two of them after the archaeological campaign in the courtyard of the Hospedería (2000, p. 54). They were large rectangular chambers with a hole in the upper part to collect falling water from the courtyards. They had several points of outflow outside their walls that allowed water to be extracted for consumption. As Castro Caturla pointed out, the cisterns were distributed in pairs to avoid the lack of water; when one was in use, the other was being replenished (1986, p. 114).

## The *Arca* de Repartimientos and the main gallery

During the years following the death of Juan Bautista de Toledo (1567), there was a period of changes in the design activity of the building which also had repercussions on the hydraulic system. More tanks and fountains began to be considered, and the eleven rainwater cisterns proved to be insufficient for the whole supply of the residence. It was at this point that the Andalusian hydraulic engineer Francisco de Montalbán came on the scene. Summoned by Philip II on 23 March 1570, he embarked on the design of a series of aqueducts to collect water from the Romeral, Helechal and Tobar streams. There is no space in this article to analyse these aqueducts, but an in-depth study of this external supply system has been recently published (Gumiel Campos 2023b). In addition, it is recommended to consult the works of Asunción De Vicente García (1991) and Jonathan Gil Muñoz (2016).

Water collected by the aqueducts designed by Montalbán flowed into the same place: the Arca de Repartimientos. This was a small two-story construction, formerly located in the square of Jacinto Benavente. It acted as the matrix of the hydraulic complex. It was the collector of the entire system of aqueducts, and the main distributor of water to the different parts of the monastery. This building was erected by the mason Simón Sánchez under the direction and designs of Francisco de Montalbán. This is certified by the contract signed on the 7th of September 1575, in which Simón Sánchez undertook to lay ‘all the stonework necessary for the water building that now begins at the entrance to the Calle de los Alamos on the Escurial side, which work has to be done according to the order given to him by Francisco de Montulban’^2^ (Bustamante García 1994, p. 309).

The Arca de Repartimientos, as described in detail in the *Libro de la Fontanería* of 1645, had eleven different points of outflow. Three of them functioned as drains from the reservoir itself, including a public fountain for the town. The other eight were different pipes distributed to eight different locations in the building. Each of them fed several fountains, tanks or other water devices.

The first shared section of the eight branches was installed in a gallery that crossed what is now Grimaldi Street and the Lonja in a N–S direction. The construction of this gallery was entrusted to the mason Francisco Rodríguez, who undertook the responsibility ‘to build the masonry in the canalisations, both of rough stone and of brick, as well as the frames and fittings of the frames, both open and closed, by the order given to him by Francisco de Montalbán’^3^ (1994, p. 309). In the same month of September 1575, the potter Gaspar de Medina was commissioned to make six hundred clay pipes one foot in diameter (30 cm) and one inch thick (2.54 cm) for the pipes to be installed in the gallery that Francisco Rodríguez was building from the Arca de Repartimientos (1994, p. 309).

In front of the northern gate of the monastery, there was a small underground tank that collected, decanted, filtered, and redistributed the water from the eight branches coming from the Arca de Repartimientos. Today, a granite slab confirms the external access to this small tank. From this tank, two of the pipes ran eastwards to supply the palace, while two others ran in the opposite direction, parallel to the wall of the building, to supply the seminary. However, the first four pipes, dedicated to the convent, were installed inside a main gallery that crossed almost the entire monastery in a N–S direction, crossing the eastern courtyards of the seminary and the Patio de Reyes, until it ended in the eastern corridor of the cloistered area.

**Fig. 3:**
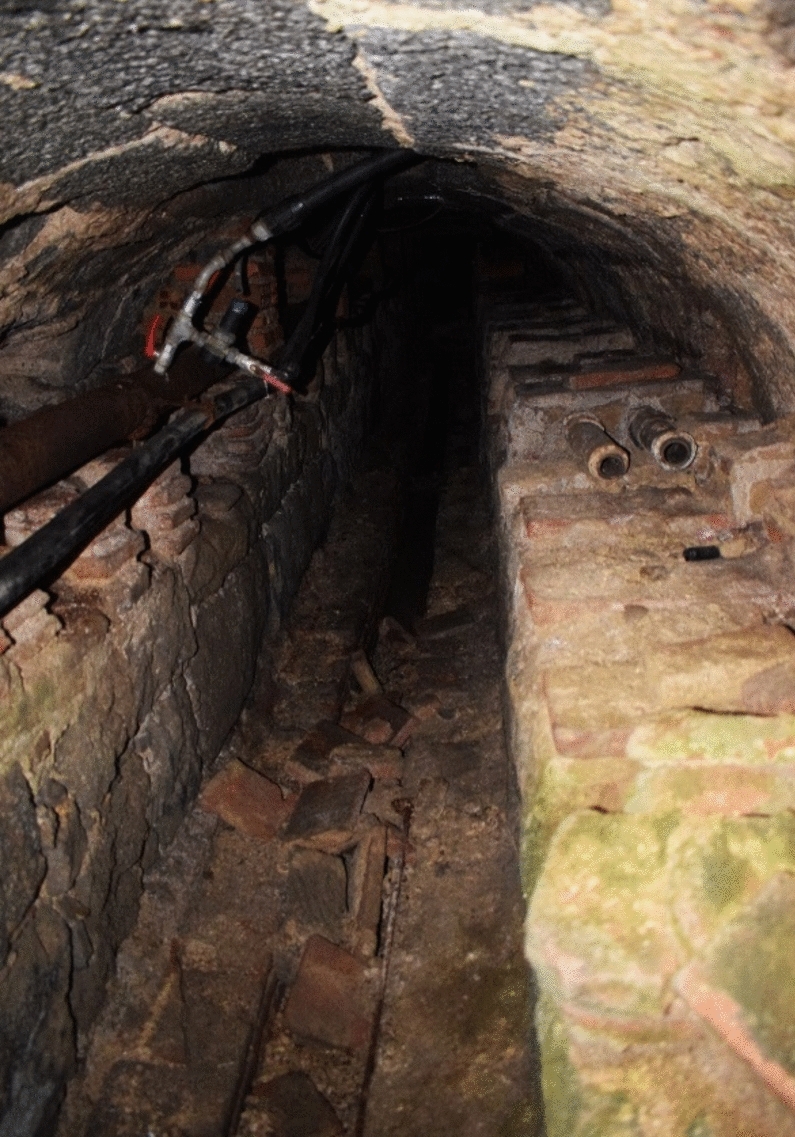
N–S main gallery. Container for the first sections of branches 1–4. Image of the author

Today, this gallery is still accessible and transitable. It has two different accesses. Firstly, a grille located in the service courtyard of the seminary at coordinates 40.5898, − 4.1478. On the other hand, a slab located in the middle of the eastern corridor of the cloistered area. As I had the privilege of seeing, this gallery has a very particular morphology (Fig. 3). It is covered by a barrel vault high enough to allow passage. On either side, there are two benches on which the first four pipes dedicated to the convent are placed. At a certain point, these four pipes of the hydraulic network bifurcate into four different areas of the convent. This bifurcation takes place in the eastern corridor of the cloistered area, around the coordinates 40.58855, − 4.14813.

### Branch 1

**Fig. 4:**
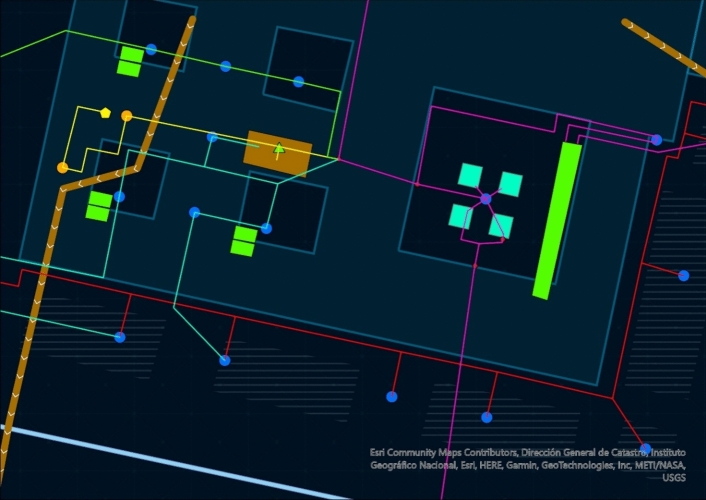
Southern area of the monastery's hydraulic network. ArcGIS Pro

**Fig. 5:**
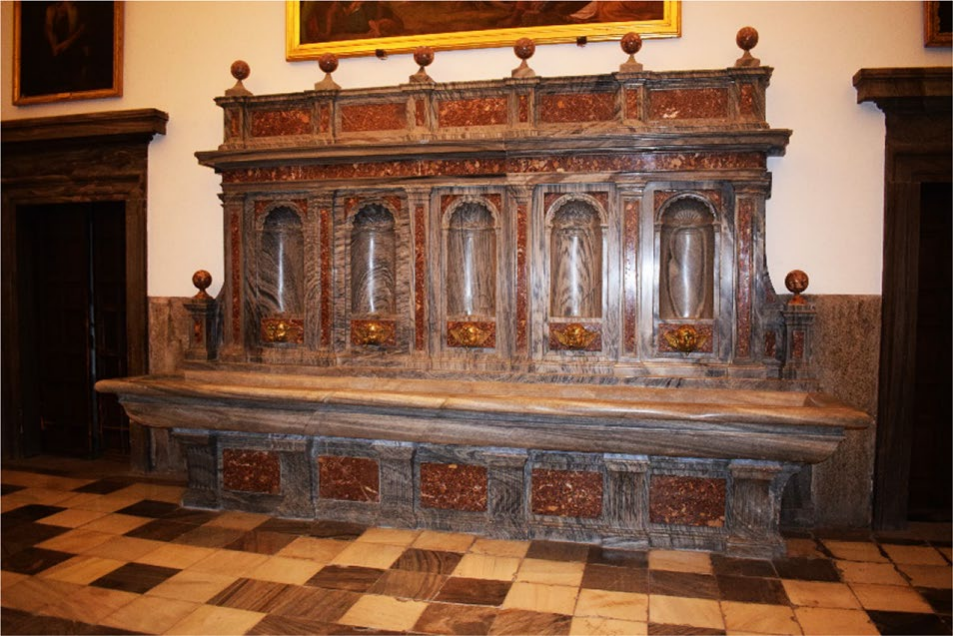
Sacristy fountain. Image of the author

**Fig. 6:**
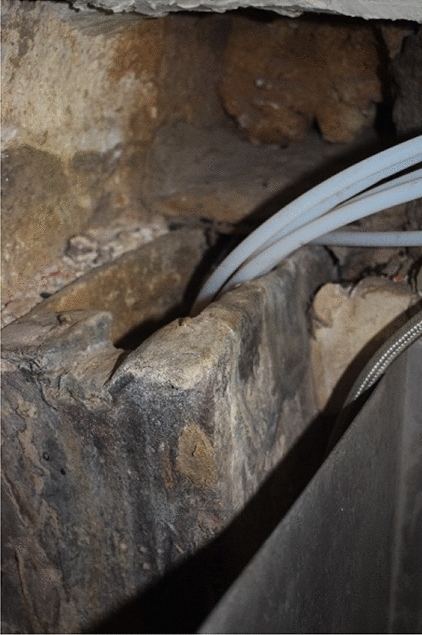
Internal channelling of the sacristy fountain. Image of the author

The first pipe distributes water essentially to the cloister of the Evangelists and the sacristy (Fig. 4: purple). Its route is explained in detail in the *Libro de la Fontanería*. The pipe forks in two before entering the main cloister. The first section leads directly into the sacristy fountain (De Andrés 1965, p. 299). It is a marble and jasper piece composed of five niches framed by Doric pilasters, and five bronze points of outflow in the shape of winged putti through whose mouths water is expelled (Fig. 5). In the room next to the sacristy, the original pipework distributing water to the five points of outflow can still be seen (Fig. 6). The fountain can be dated around 1581. At that date, Pedro Banelo signed a contract committing himself to carve jasper stones for the fountains in the sacristies of both the convent and the seminary (De Andrés 1972, p. 91). The water surplus from the sacristy fountain was directed to the large pluvial cistern located under the Evangelists cloister and from there to the eighth fountain in the garden.

The second bifurcated conduit of this first branch supplied the fountains of the Evangelistas pavilion and its four pools. Much has been written about this fountain. Its architectural morphology has led many authors to look for precedents and establish formal relationships with other contemporary buildings. Teodoro de Anasagasti (1923, p. 177) noted the influence of Bramante's pavilion in San Pietro in Montorio. George Kubler (1983, p. 7) pointed out the similarities between this fountain and the one in the cloister of Manga in Santa Cruz de Coímbra. Finally, Carmen Toribio Marín (2015, p. 304), proposed the origin of its morphology in the peninsular Islamic tradition, and insisted on the impact it had on sixteenth-century gardening, especially in cases such as the villa Lante, Saint Germain-en-Laye, or villa Gamberaia.

The construction of the Evangelistas fountain took eight years. On 22 December 1586, a contract was signed with the master stonemasons Bartolomé de Elorriaga, Pedro Castelo, Juan Antonio Marosa and Francesco Aprile for the materialisation of the fountain and its pools (Bustamante García 1994, p. 530). A few months later, a payment to the last two was recorded for extracting marble from the quarries of Estremoz in Portugal (De Andrés 1972, p. 161). Between 1587 and 1588 four other payments to Bartolomé de Elorriaga and Pedro Castello are registered for carving and placing the same marble in the pavilion and the cloister pools. In 1589 the plumber Jorge Enríquez received a stipend for five brass keys for the spouts of the pools. Finally, the icing on the cake was provided by the sculptor Juan Bautista Monegro, who in 1589 undertook to make the eight statues of the evangelists and their symbols in his workshop in Toledo from Genoese marble (1972, p. 178). These sculptures were not finished until 1593.

**Fig. 7:**
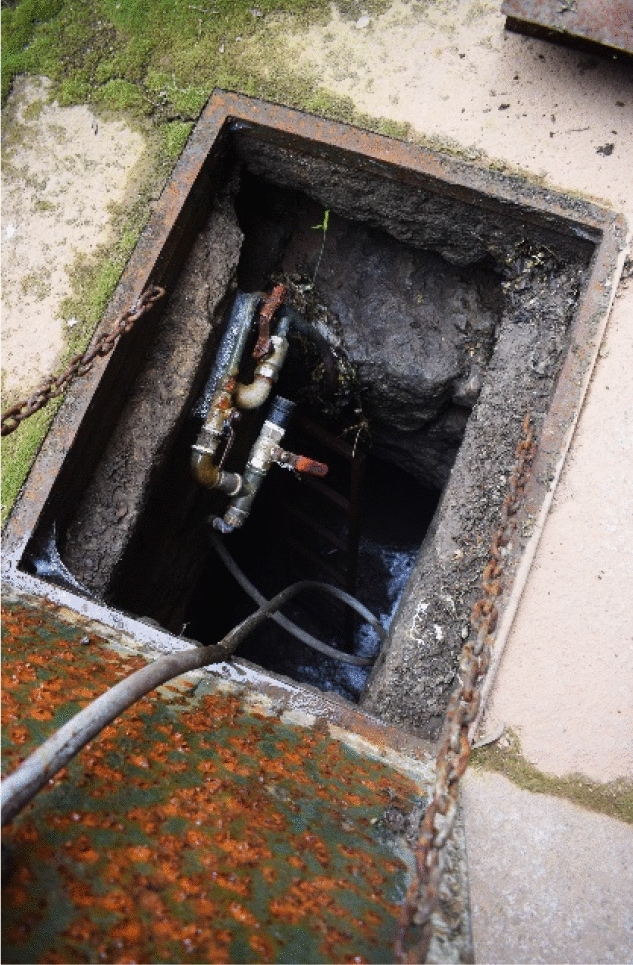
Water deposit in the courtyard of Evangelistas. Image of the author

The water surplus from the pools of the fountain of the Evangelists was used to irrigate the cloister gardens and later the orchard. During fieldwork I had the opportunity to see this underground canalisation through a small deposit located at coordinates 40.58828, − 4.1477 (Fig. 7). This irrigation channel was not recorded either in the Plumbing Book or in the maps of Kubler and De Andrés. However, it was mentioned by Juan Alonso de Almela in 1594: ‘there is a waterspout that leads to each of the said four pools or water receivers, from where these gardens are irrigated, and the water surplus from them goes through certain aqueducts to the orchard of the said house’^4^ (1962, p. 49).

### Branch 2

Returning to the bifurcation point of the first four branches located under the eastern corridor of the enclosure, let us analyse the second pipe (Fig. 4: light blue). This second pipe supplied the southern part of the convent enclosure, including the fountains of the two courtyards of Legos and Enfermería, the hallway, the refectory and the apothecary's building. Like branch 1, shortly after it became independent, the second pipe split in two parts. The first section ran to the central fountain in the cloister of Legos. From there, the water surplus fed another lost fountain located in the refectory, and from there, the pipe sent the water surplus to the third fountain in the garden of Frailes (De Andrés 1965, p. 300).

**Fig. 8:**
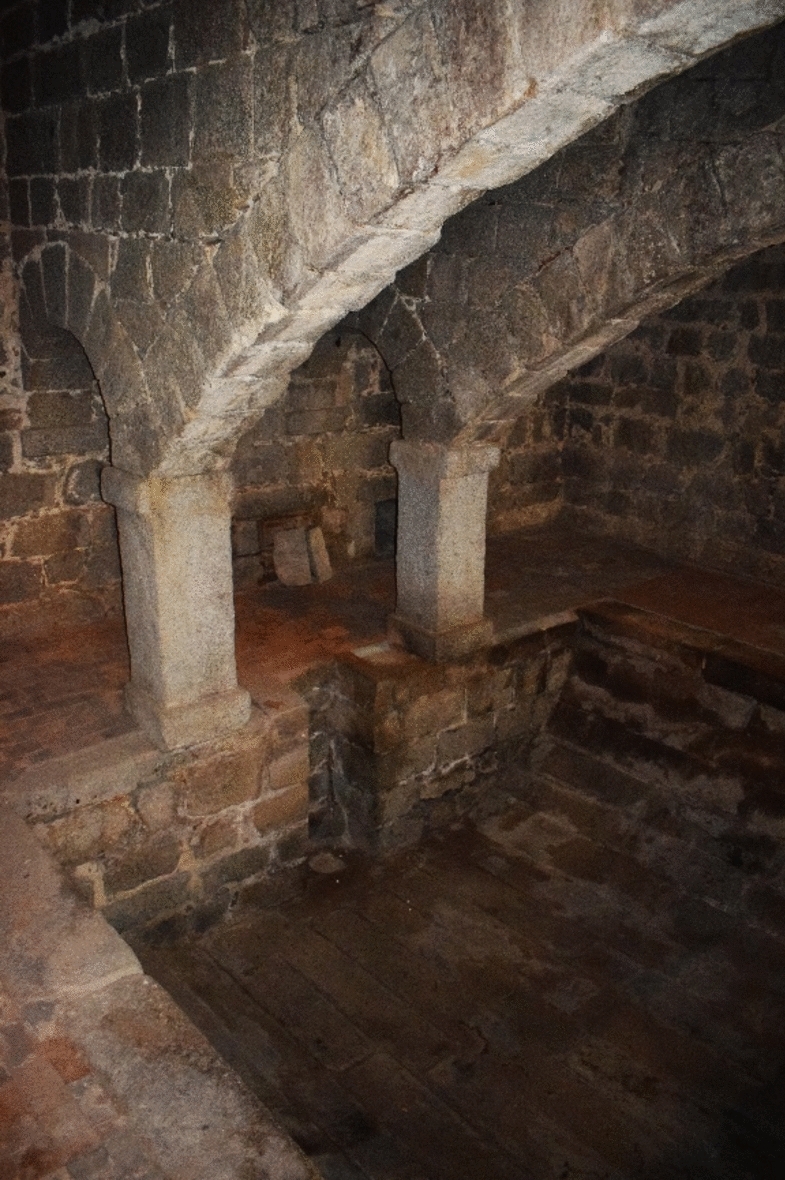
Wastewater deposit (balsa) of the convent. Image of the author

The second side of branch 2 led to the fountain in the centre of the courtyard of Enfermería. But before reaching it, there was another small diversion that separated water towards the fountain in the hallway. In turn, the surplus from this fountain was sent to a sewer known as the ‘balsa’ (1965, p. 301). A huge tank of 14 × 7 m covered by a vault supported by two basket-handle arches, which acted as a residual deposit, before sending the water to the sewers (Figs. 4: brown rectangle, Fig. 8) (De Castro 1986, p. 116). The fountain in the hallway is not the original one. Juan de Herrera catalogued it in his summary of 1589 as a work of jasper.^5^ (1589, p. 11). Juan Alonso de Almela also described it as a jasper stone inlaid with white marble, as did Fray Francisco de los Santos a hundred years later^6^ (1698, p. 58). However, the fountain that can be seen today is made of granite.

The surplus from the fountain in the courtyard of Enfermería was directed to the Galería de Convalecientes, where it fed three new points of outflow. The first, known in 1645 as the ‘fuente de la botica’ (De Andrés 1965, p. 301), served the apothecary's shop. The second, according to the description, was located next to the latrines of the apothecary's shop. Finally, the third, was in the ‘vault where the syrups were made’ (1965, p. 301). None of these three points of outflow can be traced today, but at least one of them could be associated with the fountain located on the lower floor of the Galería de Convalecientes at coordinates 40.58832, − 4.14947.

Thanks to the documentation preserved in the archive, catalogued by De Andrés in 1972, can be known how the pipes of this second branch were installed at the end of 1576. On 22 May of that year, the master stonemason Francisco Rodríguez signed a contract to lay the pipes to the infirmary under the supervision of Francisco de Montalbán. However, the construction of the two fountains of the minor cloisters of Legos and Enfermería, in addition to the fountain in the hallway, was done in 1580 by the masons Diego Serna and Pedro de Velayos (De Andrés 1972, p. 87).

### Branch 3

The third water pipe fed the two northern courtyards of the convent: Portería and Hospedería (Fig. 4: light green). The canalisation starts from the same place as its counterparts and runs to the central fountain in the Portería courtyard. From there the water flowed to an underground cellar. The *Libro de la Fontanería* mentions only one fountain in the cellar, but Juan Alonso de Almela's detailed description speaks of three^7^ (1962, p. 65). From that cellar fountain, water was raised again to the central fountain in the Patio de la Hospedería. Its surplus ran a long way under the Lonja until it reached a small tank for public use in the Calle de los Arcos, nowadays preserved outside the monastery.

**Fig. 9:**
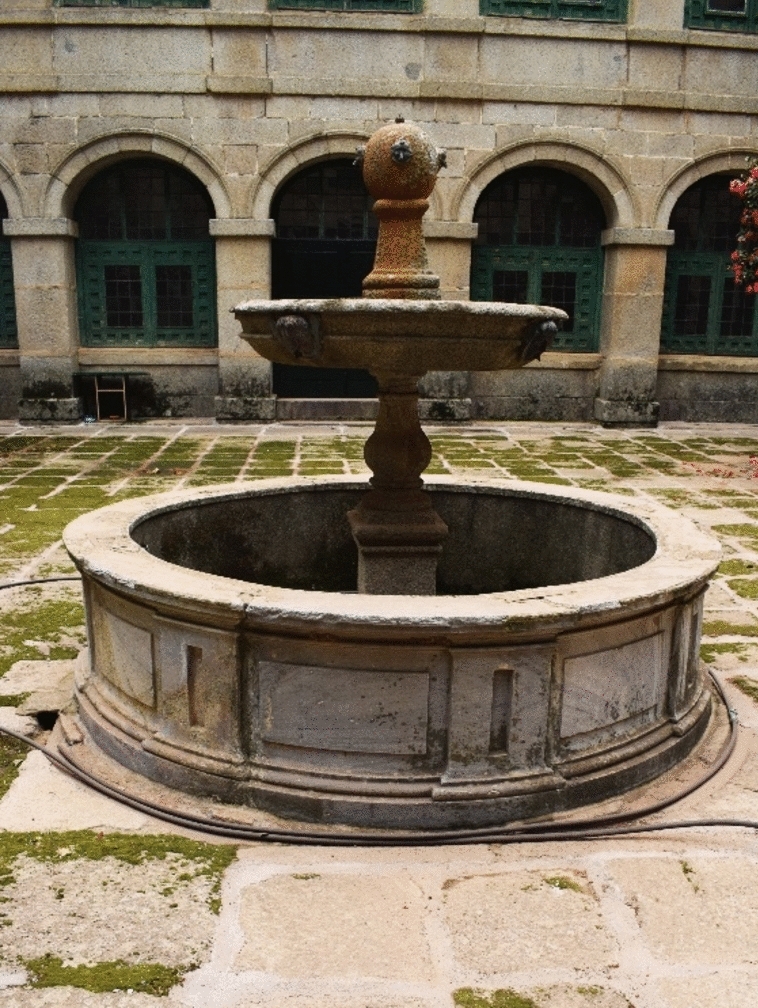
Fountain in the courtyard of Portería. Image of the author

The pipes of this third water branch were the second earliest in the building. In 1572, the Flemish silversmith Mateo de Lemiguete was commissioned to make brass pipes for the new fountains of the convent (De Andrés 1972, p. 26). In 1573 the potter Gaspar de Medina was paid for 297 clay pipes for the same waterworks (1972, p. 28). However, considering that the aqueduct was only completed in 1575, it is possible that water did not run through branch 3 until that date. Even earlier is the design (not the construction) of the two main fountains in the courtyards of the Portería and the Hospedería (Fig. 9). On 7 April 1570, Andrés Almaguer, supervisor of the monastery works, sent a letter to Martín de Gaztelu, secretary, and adviser of the king, asking Juan de Herrera to send the designs for these fountains, as the prior had demanded.^8^ (Portabales Pichel 1945, p. 260). Fray José de Sigüenza (2010, p. 116) explains how these fountains were originally made of granite but were remade and monumentalised in marble. This transformation had already been carried out during King Philip's visit in 1587.

### Branch 4

The last branch of water supplying the convent area of the Monastery fed the kitchens, the latrines, and the fish storehouse (Fig. 4: yellow). The latrines (Fig. 4: green triangle) were located on the upper floor, and as explained in the *Libro de la Fontanería*, to reach this height the water was channelled through narrow bronze pipes (De Andrés 1965, p. 303). Moreover, these narrow pipes generated water at high pressure, which was essential to avoid bad smells. As Juan Alonso de Almela described, there were two fountains on each side of the latrines connected by a channel that ran under the 16 toilets to clean the dirt. The wastewater fell directly into the ‘balsa’ on the lower floor (Almela 1962, p. 65).

Before entering the latrines, water from branch 4 was directed to the kitchen through another pipe (Fig. 4: orange circle). The kitchen was an 18 × 9 m chamber with a total of seven points of outflow. Two were in niches on either side of the entrance^9^ (Almela 1962, p. 63). Once inside, three other pipes, also located in niches in the wall, poured cold water^10^ (1962, p. 64). But most interesting was the hot water system implemented. It consisted of a boiler next to the ovens that heated the pipes^11^ (1962, p. 64). From the kitchen, water ran directly to the second kitchen of the convent, dedicated to the infirmary. A single point of outflow was installed there. Finally, returning to the main kitchen, the water surplus was drained to the underground floor where the fish storehouse was located (Fig. 4: yellow pentagon). In the fish storehouse, there were five tanks where fish was kept (1962, p. 64).

By comparing the preserved historical sources, two construction phases can be discerned in branch 4. On the one hand, there is a contract from the year 1566 to build a pipe from the kitchen to the latrines of the convent (De Andrés 1972, p. 12). But bearing in mind that the aqueduct only was ready in 1575, this pipe had to be fed in another way. In fact, there is a letter from the prior of the monastery to Pedro del Hoyo, secretary of the king, dated 2 November 1565,^12^ in which he proposes to redirect rainwater to the latrines of the convent (Portabales Pichel 1945, p. 218). Therefore, it is possible that these first pipes built around 1566 were supplied by the rainwater cisterns. These would have been the first pipes in operation in the whole building, which makes sense considering that they were intended for the kitchens and latrines, essential parts for the monk`s habitability.

However, it seems that from 1570, when Francisco de Montalbán designed the aqueduct, the system for supplying the kitchens and latrines was reformulated by means of the new water branch 4. On 28 January 1571, the prior Fray Hernando de Ciudad Real wrote a letter to the king, informing and being sceptical about the installation of the kitchen heating system.^13^ (Portabales Pichel 1952, p. 118). Another letter dated 11 February 1573 certifies how the (new) latrine pipes were being finished (Bustamante García 1994, p. 273). Finally, in 1582, the pipes under the fish storehouse and the latrines were tiled and covered with cobblestones (De Andrés 1972, p. 105).

### Branch 5

**Fig. 10:**
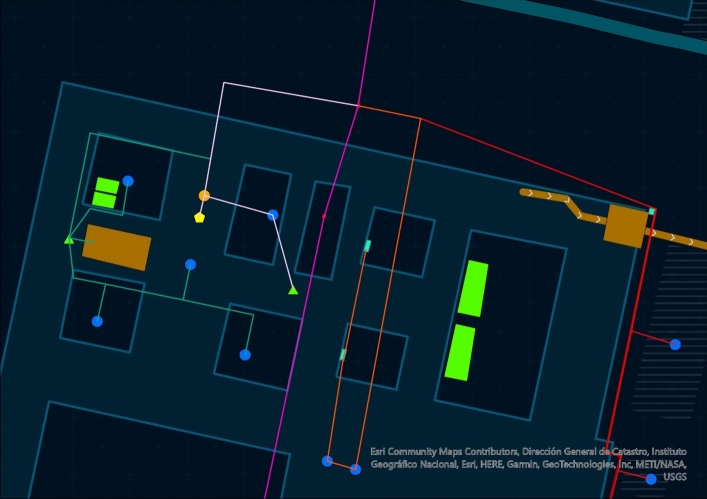
Northern area of the monastery's hydraulic network. ArcGIS Pro

Having analysed the route of the first four pipes that ran through the main gallery, let us return to the starting point to understand the distribution of the other four branches. Branch 5 started from the tank located at the northern gate of the monastery and was directed towards the east to supply the church, the sacristy of the seminary and the kitchen of the palace (Fig. 10: orange). Some slabs are still preserved in the first courtyard of the palace at coordinates 40.58969, − 4.14757 indicating the transit of this pipe. The pipe crossed both small courtyards to reach the church. There, through narrow bronze pipes, it ascended thirty feet (9 m) to the first floor, where the choir fountain was located (De Andrés 1965, p. 303). From the choir fountain, water flowed to the fountain in the sacristy of the seminary, now lost, but originally located next to the staircase. After that, it was channelled back to the small courtyards of the palace, feeding the two small tanks and the king's kitchens (1965, p. 305). The plan of the building in Herrera's designs depicts two fountains, but only one of them remains today.

The only historical source that could help us to date this branch 5 is the aforementioned contract with the mason Pedro Banelo to carve jasper stones for the fountains of the sacristies of the convent and seminary in 1581 (De Andrés 1972, p. 91). But I could venture that, according to the construction dates of the seminary and the choir of the church itself, pipe number five was one of the last hydraulic devices to be installed in El Escorial.

### Branch 6

**Fig. 11:**
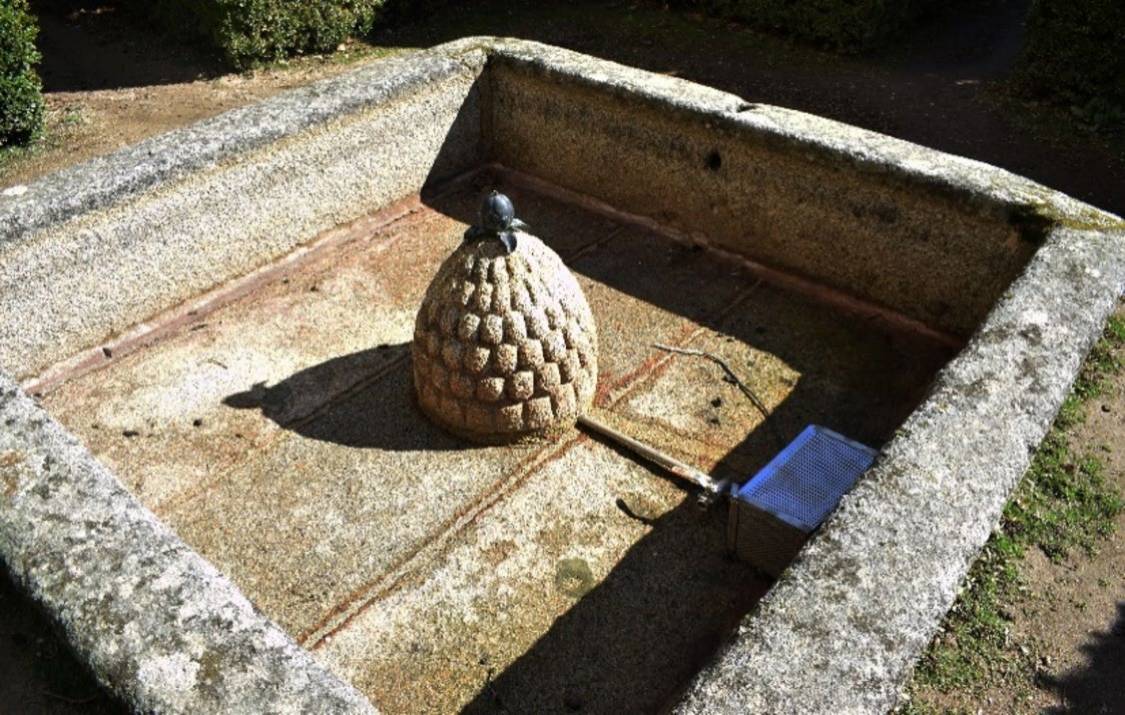
Fountain in the Garden. Image of the author

This pipe was dedicated to the gardens and the courtyard of Mascarones (Fig. 10: red). The pipe runs, together with the fifth branch, from the small reservoir to the tower of the Ladies located in the northeast corner of the monastery. A small deposit was installed there, which probably generated pressure for the pipe. From that arc, the pipeline irrigated each of the twelve fountains in the garden surrounding the monastery on its east and south flanks. The pipe ran parallel to the monastery wall sending water through short conduits that saw the light in the jets of the twelve fountains (De Andrés 1965, p. 306). The colossal length of branch 6 is remarkable, and it seems complicated to supply all the devices. But let us remember that the first three fountains on the southern flank of the garden were also supplied through the surplus of branch 2. It could even be argued that, because of a possible hydraulic insufficiency in the first fountains of the garden, an extension of branch 2 was considered to satisfy them. The morphology of the garden fountains is very simple (Fig. 11). The water is expelled through a granite pinecone, falling into a square container of about three metres on a side. The surplus was used to irrigate the gardens and then the orchard.

**Fig. 12:**
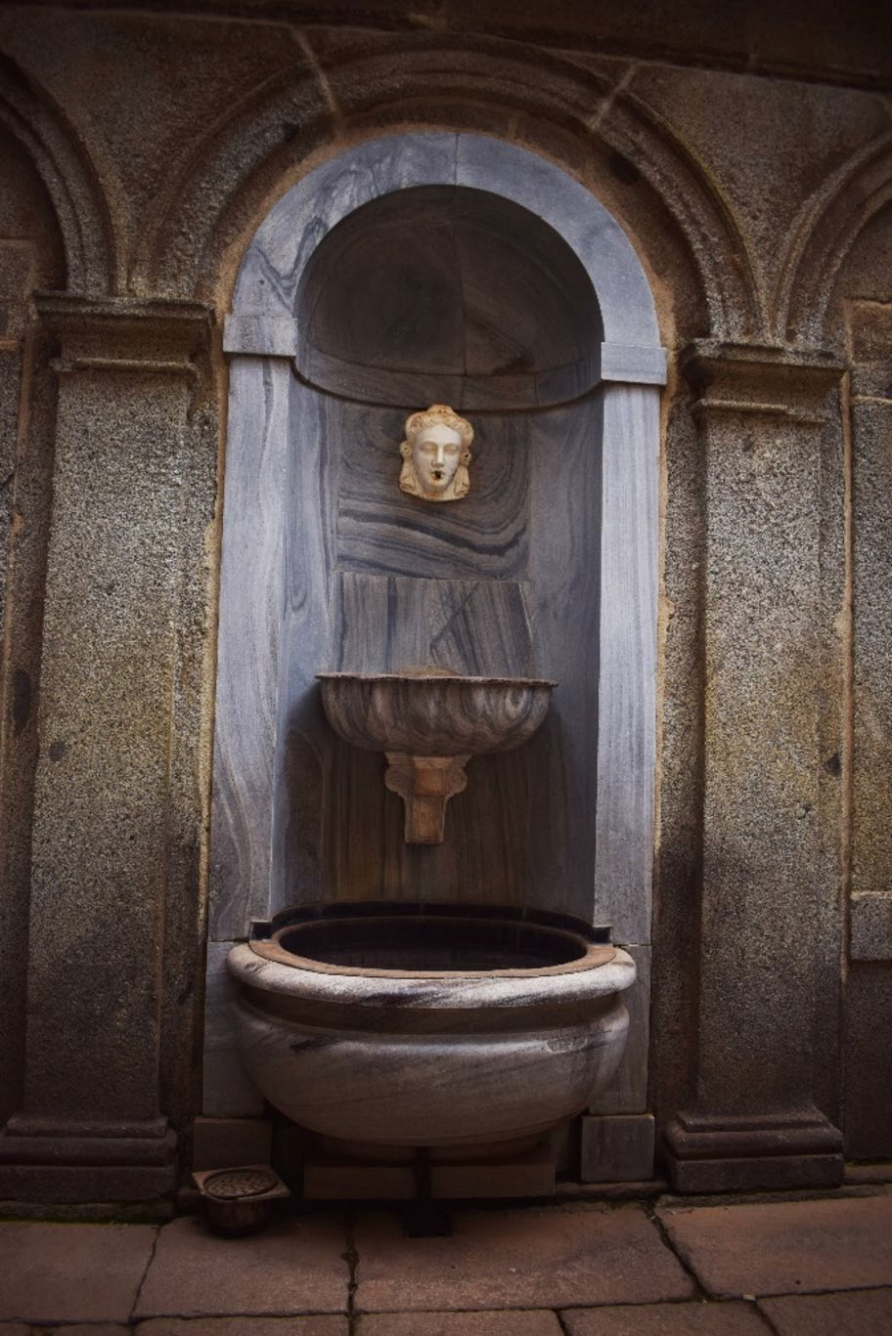
Southern fountain of the courtyard of Mascarones. Image of the author

From the small deposit in the tower of the ladies, a second pipe is used to feed the palace of Philip II (1965, p. 306). In the courtyard of Mascarones, two feminine-masked fountains are framed inside two marble niches (Fig. 12). Although the 1645 description does not explain more about this sixth pipe, the lack of other hydraulic devices in the private palace seems strange. In fact, Juan Alonso de Almela tells us about other fountains in the royal residence for cleaning purposes^14^ (1962, p. 79). This sixth branch was probably installed between 1573 and 1576. There is a letter dated October 4 of the first year, explaining how the designs for the garden fountains are ready (Bustamante García 1994, p. 273). Three years later, the bricklayer Hernando de la Cruz signed a contract to build the walls, pipes, and drainage galleries of the southern garden (De Andrés 1972, p. 55).

### Branch 7

The supply for the convent and the palace has been analysed, so the only missing part is the seminary. From the small deposit located in front of the northern gate, two other conduits were diverted westward along the north side of the seminary. The first (Fig. 10: white) supplied the seminary´s kitchen, where another boiler with identical characteristics to the one in the convent was installed. Following the same scheme, from the kitchen, water also slid to the fish market, which was on the lower level (De Andrés 1965, p. 307). There was a secondary branch, from the kitchen to the so-called ‘wood yard,’ which probably refers to the northeastern service yard of the seminary. In said patio water fed a fountain formerly located in its center. Currently there is no fountain in this space, but Juan Alonso de Almela made it clear that each of the patios of the seminary had a fountain with characteristics similar to those of the convent^15^ (1962, p. 73). Water was later sent to the palace's latrines, which, as can be seen in Herrera's designs, were located behind the refectory (De Andrés 1965, p. 308).

### Branch 8

The last of the pipes fed the rest of the water devices of the seminary. First, it was raised to the second floor to supply the latrines, which had an identical hygiene system to the convent. From the latrines, a water diversion fed the fountains of all the courtyards and the central hall. Residual waters from the latrines and the fountains were sent to another sewer deposit (*balsa*) under the latrines (De Andrés 1965, p. 307). It is complicated to date the hydraulic infrastructure of the seminary. Unfortunately, there are no contracts or testimonials preserved. However, taking into consideration the constructive evolution of the monastery, being the seminary one of the last parts to be built, it is reasonable to say that the seventh and eighth branches were the last to be installed.

## The three sewers

A large part of the water surplus from the eight pipes fed the garden and orchard of the monastery. However, another significant amount of wastewater was stored in the ‘balsas’ and had to be expelled from the building to guarantee optimal sanitary conditions. For this reason, three large sewers were built. The estimated route is represented in Fig. 2 with a brown line with arrows.

**Fig. 13:**
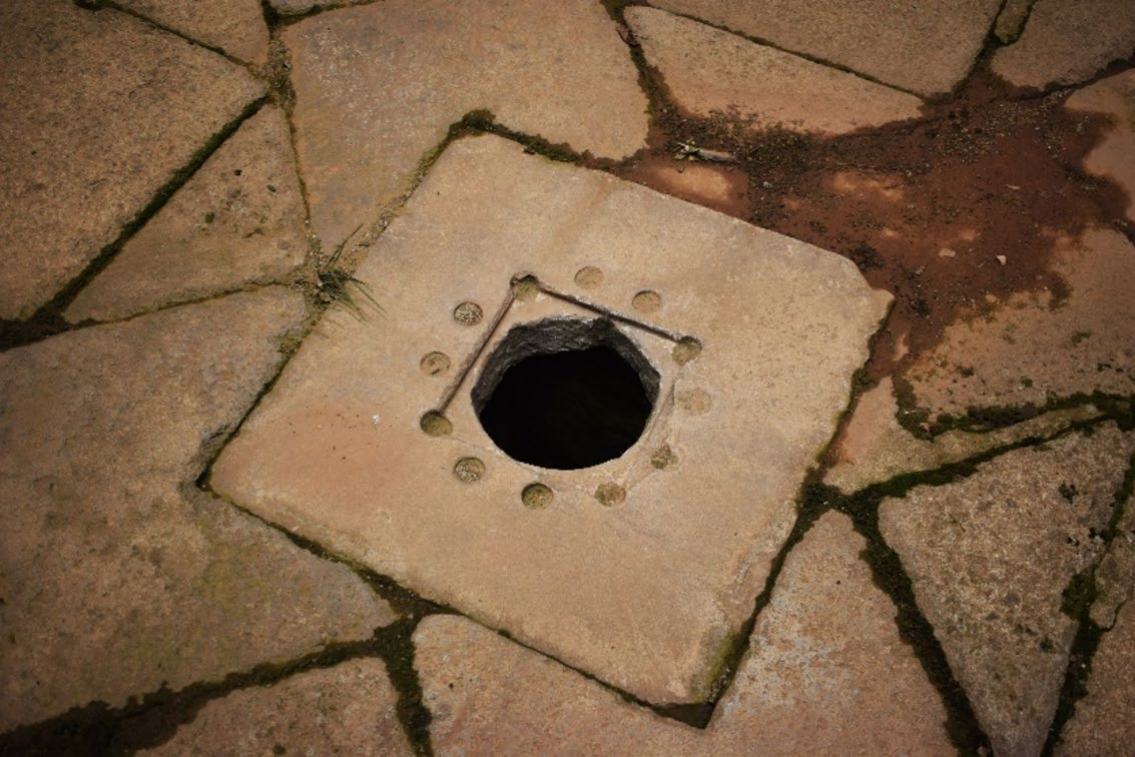
Drain in the centre of the courtyard of Mascarones. Image of the author

The main sewer, although not the first, was studied in 2013 by Chías Navarro and his team after performing a georadar campaign that unearthed some of the underground sections (Chías Navarro et al. 2013, p. 177). This main sewer crossed the basement of the church, the royal pantheon, and the garden with a W-E orientation with a slight southern inclination. The drainage channel saw the light in a primitive pond known as the ‘Bosquecillo’. This sewer received wastewater from the main hydraulic devices on the eastern side of the monastery, that is, the church, part of the fountains in the cloister of Evangelistas, and the fountains in the courtyard of Mascarones, in whose floor the drain is still preserved (Fig. 13).

**Fig. 14:**
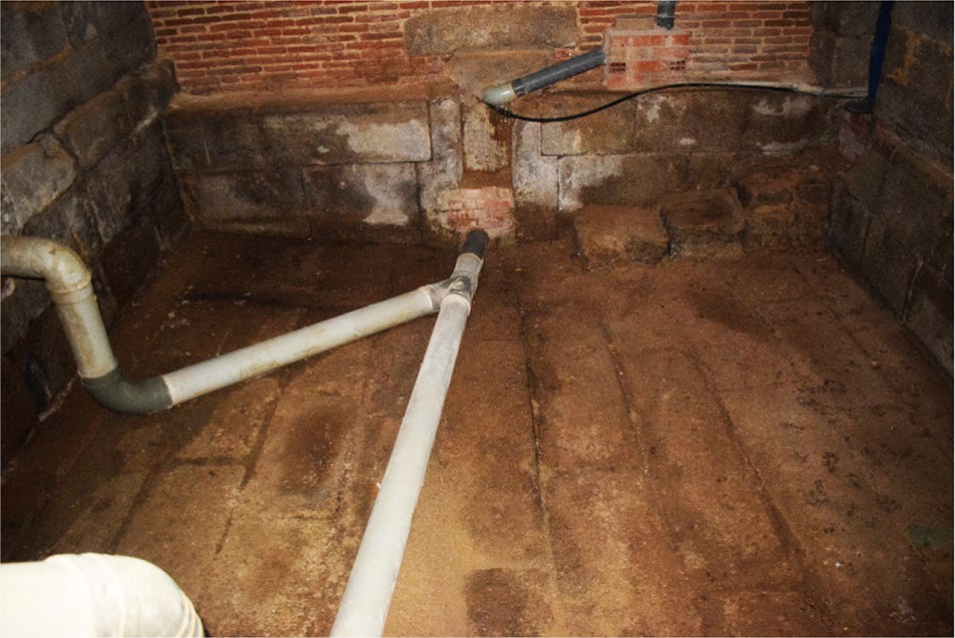
Wastewater deposit (balsa) under the Torre de las Damas. Image of the author

**Fig. 15:**
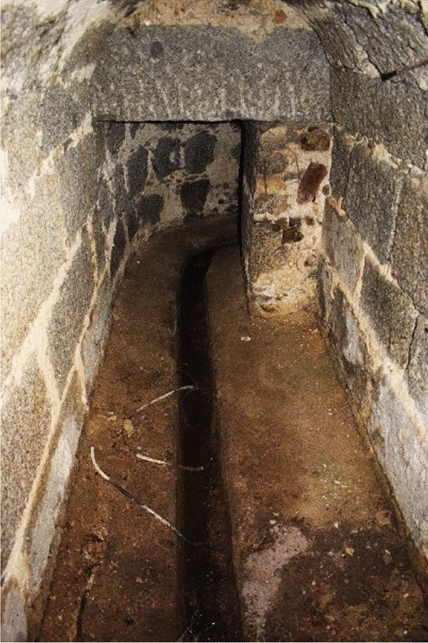
Canalización de la cloaca bajo la torre de las Damas (imagen del autor)

The second sewer of the monastery was constructed under the northeast tower, the Torre de las Damas. It was well described by the *Libro de la Fontanería*^16^ (De Andrés 1965, p. 310). I have had the privilege of entering the ‘balsa’ and observing that it has a similar morphology as the deposits located under the convent and the seminary (Fig. 14). This deposit was fed by a sewage pipe coming from the west (Fig. 15), which probably collected waste from the kitchens and latrines of the palace. As the *Libro de la Fontanería* explains, another conduit started from the waste deposit, now blocked off, which ran east through the queen's garden.

The last sewer was located under the cloisters of the convent's closure and collected most of the monastery's wastewater. There is a letter written in 1692 that warns about the risk of flooding of this third sewer in which its morphology is clearly described. It is explained that it collected water from the kitchens, the latrines of the convent and the seminary, the infirmary, the pharmacy, the Patio de Reyes, and rainwater from the Lonja^17^ (De Andrés 1965, p. 316). In other words, all the surplus from western water devices that was not used to water the gardens and orchard.

A lot of historical documentation is preserved about the construction of this third sewer. In fact, it was probably the first one built. On April 19, 1566, Juan de la Huerta and Toribio de Escobedo signed a contract to work on the main sewer that goes under the kitchen and the latrines until it goes out of the building^18^ (Bustamante García 1994, p. 148). On April 28, a payment of 5330 reals is granted to Toribio de Escobedo for his work on the sewer, and on June 21, Miguel Sánchez received another payment of 5594 reals to cover his vaults (1994, p. 148). Chías Navarro also discovered a small hand sketch by Juan Bautista de Toledo, which represents this third (or first) sewer under the convent kitchen (Chías Navarro 2013, p. 40).

## Brief comparative analysis and conclusion

The construction of the hydraulic system of the Escorial is framed in a historical period in which water had already become a priority in residential architecture. During the second decade of the sixteenth century, there were already many residences both Iberian and European that had a powerful supply system, thought to satisfy the increasing needs of their inquiline`s water consumption. Philip II himself commissioned other colossal hydraulic works. He built immense dams or ponds for navigation and recreation in the Casa de Campo (Navascués et al. 2009) or Aranjuez (Miguel Rodríguez and Segura Graíño 1998). In Portugal great water infrastructures were also built in the National Palace of Sintra (Gumiel Campos 2023a), the Ducal Palace of Vila Viçosa (Gumiel Campos 2023c), the Quintas da Bacalhôa, Torres, and Ribafría (Duarte Rodrigues 2019), not to say the magnificent aqueducts projected all along the century in Évora, Elvas or Tomar (Tchikine 2020). Outside the Iberian Peninsula, can be mentioned the water displays projected in Boboli (Lamberini and Tamantini 2013), Este, Caprarola, Bagnaia (Esposito 1986), Pratolino (Zangheri 1978), Fontainebleau (Droguet 2012), Hellbrunn (Bigler 1996), Heidelberg (Gensichen 2020) and many others palaces all along the continent.

It can be therefore argued that the hydraulic infrastructure of El Escorial is nothing new, but the same construction principles in vogue in all the continent are certainly taken to large scale in this building. The infrastructure is greater than in many other of the mentioned residences, as were the costs involved. After all, everything is a response to the promotion of the main building of one of the most powerful monarchs of sixteenth-century Europe, who in addition was a real enthusiast and promoter of architecture. It is also interesting to see how despite the complexity of the water supply system, none of the waterworks that populated El Escorial were too ostentatious. Unlike in the papal courts or the Medicean villas, there are no automata, *grottoes*, or flooded banquets. This cannot be attributed to a lack of information or funds. This was a deliberate decision by the promotor, whose more sober and austere character influenced the design of El Escorial.

## Footnotes

1 The original letter prays: *‘*Next to each of the cisterns there is to be a small chamber, and in the wall between it and the cistern there is to be a point of outflow through which they take the water, not from the lowest, nor the highest, but from the middle one, which is cleaner*’*. [‘Junto a cada una de las çisternas ha de hauer vna camarilla, y en el muro que esta entre ella y la cisterna ha de hauer vna llave por donde tomen el agua, no de la mas baxa, ni de la mas alta, syno de la de medio que es mas limpia’].

2 Archivo de la Biblioteca de San Lorenzo de El Escorial (ABE), IV-17. ‘toda la piedra labrada que fuere menester para el edificio del agua que aora se empieça a la entrada de la calle de los alamos a la parte del Escurial la qual obra a de azer por la orden que le diere Francisco de Montulban’.

3 ABE, IV-17. ‘a azer la albañiria en la dicha obra desta encañadura asi de piedra tosca como de ladrillo y encañaduras y guarniciones de encañaduras asi de canos abiertos como cerrados la qual obra a de azer por la orden que le diere Francisco de Montalbán’.

4 ‘sale un caño de agua que da en cada uno de los dichos cuatro estanques o recibidores de agua, de donde se riegan estos jardines, y las aguas superfluas de ellos van a parar por ciertos acueductos a la huerta de dicha casa’.

5 ‘ay en medio della una gentil fuente de jaspe’.

6 ‘En medio del suelo tiene una fuente de jaspe colorado y embutidos de mármol blanco’.

7 ‘además de que hay dentro de la dicha bodega otras tres fuentes para su ministerio del agua’.

8 Archivo General de Simancas (AGS), Obras y Bosques, Escorial, leg. 6. ‘quisiera su Paternidad que Herrera le embiara también la traça de las fuentes de los dos claustros pequeños que le pidio porque se han de enlosar y seria vien que estuvisen las fuentes asentadas cuando se enlosaren’.

9 ‘Tiene a los dos lados, dos fuentes metidas en la pared con sus canillas y tornillos de bronce, como todas las demás fuentes de la casa’.

10 ‘hay además en el dicho cuerpo de la cocina otras tres fuentes o caños de agua fría para su ministerio metidos en la pared a forma de hermosas fuentes’.

11 ‘Tiene también esta gran cocina una gran caldera arrimada al horno, que caben más de cincuenta cantaros de agua, que con el calor del fuego de la cocina y el que a ella en particular se le da, está siempre el agua caliente para el servicio de la cocina; la cual da el agua a un lado por dos caños o canillas con sus tornillos bien calientes’.

12 AGS, Obras y Bosques, El Escorial, leg. 2, fol. 163.

13 AGS, Obras y Bosques, Escoria, leg. 4. ‘En lo que toca a la orden que se trata para calentar el agua com la campana o caño de bronce debaxo del fuego, si saliese çierto sería muy buen ingenio, pero parece difícil’.

14 ‘además de las fuentes que para el aseo y limpieza de ella tiene, como toda la demás máquina dicha’.

15 ‘Tiene cada uno de estos dos patios su fuente en medio, de la hechura y materiales que las de los cuatro patios del convento’.

16 ‘muchos remanentes y agua llovediza van a la torre de las damas a la balsa que allí hay; y cuando quiera que aquello se limpia, va por un conducto y atraviesa el jardín de levante y bajando por entre las dos escaleras que allí hay, sale al bosquecillo y camina derecho al Escorial’.

17 ABE, leg 109.

18 ABE, I-66. ‘dar un destajo en la madre principal con caño que ba por debajo de la cocina desde las necesarias hasta salir fuera’.
